# Effect of a self-assembling peptide hydrogel on delayed bleeding following endoscopic sphincterotomy: Prospective pilot cohort study

**DOI:** 10.1055/a-2803-3921

**Published:** 2026-02-25

**Authors:** Yusuke Ishida, Naoaki Tsuchiya, Takehiko Koga, Takanori Kitaguchi, Keisuke Matsumoto, Makoto Fukuyama, Kaori Hata, Kei Nishioka, Noriko Shiga, Tsutomu Iwasa, Hiroto Ishikawa, Ryohei Nomaru, So Imakiire, Hiroki Matsuoka, Nobuaki Kuno, Sadahiro Funakoshi, Shinya Ashizuka, Eiji Sadashima, Fumihito Hirai

**Affiliations:** 1 38068 Department of Gastroenterology and Medicine, Fukuoka University Faculty of Medicine, Fukuoka, Japan; 2 91358 Department of Gastroenterology, Saiseikai Futsukaichi Hospital, Chikushino, Japan; 3 73486 Department of Surgery, Munakata Suikokai General Hospital, Fukutsu, Japan; 4 Department of Medical Research Institute, Saga-Ken Medical Centre Koseikan, Saga, Japan

**Keywords:** Pancreatobiliary (ERCP/PTCD), Stones, Strictures, Quality and logistical aspects, Performance and complications

## Abstract

**Background and study aims:**

Endoscopic sphincterotomy (EST) is important in endoscopic retrograde cholangiopancreatography, but bleeding remains its common complication. This pilot study evaluated the efficacy and safety of a self-assembling peptide hydrogel (SAPH; PuraStat) in managing EST-related hemorrhage.

**Patients and methods:**

A prospective cohort study was conducted from June 2023 to March 2024 at three hospitals in Japan, enrolling patients undergoing EST. Patients were divided into SAPH (received SAPH for EST-related bleeding) and control groups (patients without EST-related hemorrhage); primary endpoint was incidence of delayed bleeding.

**Results:**

Of the 254 patients analyzed, 27 were in the SAPH group and 227 in the control group. Background factors related to bleeding were aligned using propensity score matching (PSM). Incidence of EST-related bleeding was 10.6% (27/254). In the SAPH group, 26 of 27 patients (96.3%) achieved successful hemostasis using SAPH alone. Although no delayed bleeding occurred in this group, it occurred in four patients in the control group (1.57%, 4/254). Other adverse events showed no significant difference between the groups. Results were similar to those after PSM and in the subgroup analysis excluding those with self-expandable metallic stent placement.

**Conclusions:**

SAPH is a simple, effective, and safe hemostatic option for treating EST-related hemorrhage and may be a promising first-line approach. This pilot study did not demonstrate a significant reduction in delayed bleeding, but absence of delayed bleeding in the SAPH group is noteworthy and suggests a potential preventive benefit. Thus, larger randomized controlled trials are warranted to validate these preliminary findings.

## Introduction

Endoscopic sphincterotomy (EST) is a fundamental and crucial technique in procedures related to endoscopic retrograde cholangiopancreatography (ERCP). However, bleeding remains its most frequent complication, with a variable incidence of 0.5% to 12% 1 2 3 4 . Endoscopic treatment is considered the first-line approach for managing EST-related hemorrhage, with various methods reported in the literature 2 5 6 7 8 9 . Each of these methods has its own advantages and limitations and choice of technique often depends on endoscopist expertise or discretion.

Recently, PuraStat (3D-Matrix Europe SAS, France), a novel self-assembling and fully synthetic hydrogel peptide, was developed as a hemostatic agent and is currently used in endoscopic procedures 10 11 12 13 14 . In addition, its utility has been reported for various gastrointestinal hemostases, especially for preventing delayed bleeding 15 16 17 . However, data on use and efficacy of a self-assembling peptide hydrogel (SAPH) specifically for EST-related hemorrhage remain limited.

Therefore, this prospective pilot cohort study aimed to evaluate the efficacy and safety of SAPH in managing EST-related hemorrhage.

## Patients and methods

### Study design

This study enrolled all consecutive patients scheduled for EST between June 2023 and March 2024 at Fukuoka University Hospital, Saiseikai Futsukaichi Hospital, and Munakata Suikokai General Hospital. All included patients provided informed consent for the procedure and inclusion in the registry. The study protocol conformed to the ethical guidelines of the 1975 Declaration of Helsinki as reflected in a priori approval by the institution’s Human Research Committee. It was also approved by the ethics committee of Fukuoka University Hospital (IRB number: H21–08–006) and registered in the UMIN Clinical Trial Registry (UMIN000051301).

### Selection criteria

Inclusion criteria for this study were EST deemed as necessary and age at least 20 years. Conversely, exclusion criteria were histories of EST, endoscopic papillary balloon dilation (EPBD) without EST, and endoscopic papillectomy. Patients who underwent precut because of difficult cannulation were excluded from the analysis. In addition, patients with malignant biliary obstruction involving the ampulla of Vater or extending to the periampullary region were excluded.

### Study outcomes

The primary endpoint was incidence of delayed bleeding. Secondary endpoints included the rate of adverse events (AEs) (pancreatitis, cholangitis, or others) and clinical success rate of initial hemostasis by SAPH.

### Definitions

This study defined EST-related bleeding as hemorrhage occurring immediately after EST and hemorrhage that occurred by contact with devices for stone removal or biliary drainage. This definition was used because our primary endpoint was incidence of delayed bleeding and we intended to capture all bleeding events potentially related to EST. Patients with EST-related bleeding not exhibiting spontaneous hemostasis for 2-minute observation or persisting until the end of planned procedures underwent endoscopic hemostasis. EST-related hemorrhage was classified into the following three categories: 1) mild oozing, continuous low-volume bleeding in which the bleeding point remained identifiable after water lavage; 2) moderate oozing, more pronounced oozing in which active bleeding was present but the exact bleeding point could not be clearly visualized even after repeated water lavage; and 3) spurting, forceful arterial bleeding characterized by a pulsatile jet. Bleeding that occurred after scope removal indicated delayed bleeding. Procedure time was measured from scope insertion to scope removal. Moreover, requirement of more than 5 minutes of biliary cannulation defined difficult cannulation. Clinical success in hemostasis using SAPH was defined as hemostasis achieved using less than 3 mL of hydrogel. AEs were scored using the lexicon of the American Society for Gastrointestinal Endoscopy 18 .

### Procedures and study protocols

EST was performed using a pull-type sphincterotome (CleverCut3V; Olympus medical systems, Tokyo, Japan or Correctome; Boston Scientific, Marlborough, Massachusetts, United States), with the medium incision oriented in the 11 and 12 o’clock directions. The standard electrocautery unit (ERBE VIO200S; ERBE, Tubingen, Germany) was also used, with a setting of an effect 2 in the Endocut I mode (output limit, 155 W). Experts with 10 years of ERCP experience performed all EST procedures, although other procedures including biliary cannulation, stone removal, and stent placement were performed by endoscopists with varying levels of expertise, including trainees. In stone removal, EPBD of approximately 10 to 14 mm was added as required.

Antiplatelet agents were not discontinued in principle, but patients with antiplatelet discontinuation 2 to 3 days before EST were included in this study. We also included patients with anticoagulant discontinuation only on the day of EST. All such cases were defined as cases with antithrombotic agent usage.

**Fig. 1:**
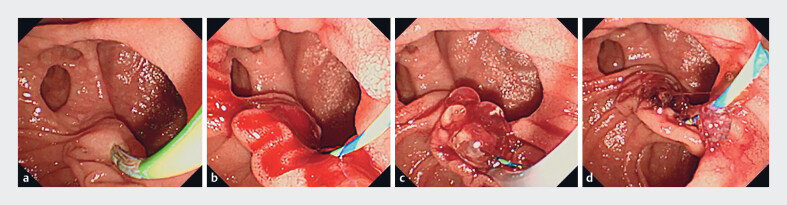
**a** Duodenal papilla before endoscopic sphincterotomy (EST). **b** Active bleeding immediately after EST. **c** Hemostasis achieved after applying a self-assembling peptide hydrogel (SAPH). **d** Sustained hemostasis after SAPH removal

Patients with EST-related bleeding who received SAPH as initial hemostasis were classified as the SAPH group. During the study period, the primary treatment strategy for EST-related bleeding was SAPH application. If hemostasis was not achieved with SAPH alone, an alternative hemostatic method such as balloon tamponade or self-expandable metallic stent (SEMS) placement was used. The hemostatic procedure using SAPH was performed in a standard manner of ERCP using a side-viewing endoscope (TJF-260V and TJF-Q290V; Olympus medical systems, Tokyo, Japan), followed by EST. SAPH was applied on the bleeding site using a dedicated catheter under endoscopic vision, with the catheter tip pressed against the bleeding point ( Fig. 1 ). The maximum amount of SAPH was 3 mL per procedure and the actual volume of SAPH used was recorded when hemostasis was achieved with < 3 mL of SAPH. The volume was calculated by measuring the residual amount remaining in the syringe after the procedure and subtracting it from the initial 3 mL. All hemostatic procedures were performed by experts with 10 years of ERCP experience. Meanwhile, patients without EST-related bleeding were assigned to the control group.

### Statistical analysis

We used Fisher’s exact test for comparing categorical data and the Mann-Whitney *U* test for continuous data. Propensity score matching (PSM) was used to adjust for differences between the two treatment groups. A logistic regression model was used for two propensity score estimations: PSM-1, which was based on patient-related factors including age, sex, use of antithrombotic agent and timing of resumption, prothrombin time-international normalized ratio [PT-INR], platelet count, liver cirrhosis presence, hemodialysis, and concomitant cholangitis; and PSM-2, which included all PSM-1 variables plus procedure-related risk factors for EST-related AEs (particularly bleeding), such as concomitant EPBD, altered anatomy, duodenal stricture, and periampullary diverticulum 1 19 20 21 . We performed one-to-one PSM between the SAPH and control groups, using the nearest neighbor method within a caliper width of 0.2 of the SD of the logit of the propensity score. Given that SEMS placement is a potential hemostatic technique 8 22 , we also compared clinical outcomes between the SAPH and control groups in the subgroups, excluding planned SEMS placement, similar to the main analysis. Statistical data were analyzed using the R software (version 4.2.2, https://www.r-project.org [accessed on October 31, 2022]). *P* < 0.05 was considered statistically significant.

## Results

**Fig. 2:**
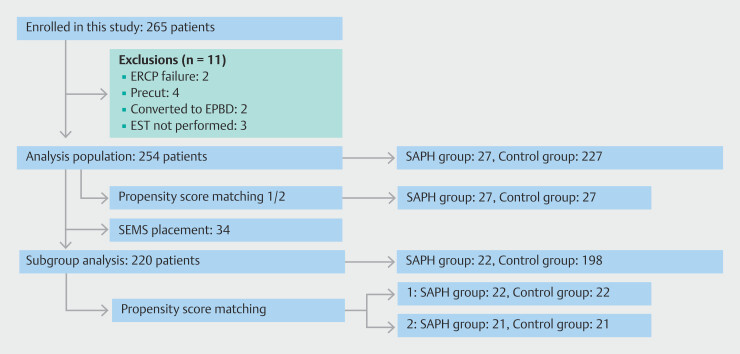
Flow diagram detailing study patients. EPBD, endoscopic papillary balloon dilation; ERCP, endoscopic retrograde cholangiopancreatography; EST, endoscopic sphincterotomy; SAPH, self-assembling peptide hydrogel; SEMS, self-expandable metallic stent.

Of 265 patients who were initially enrolled in this study, 11 (2 ERCP failure cases, 4 precut cases, 2 cases with convert to EPBD without EST, and 3 cases in which EST was not performed) were excluded, leaving 254 patients for the final analysis. The SAPH group comprised 27 patients, whereas the control group included 227 patients ( Fig. 2 ). Table 1 summarizes participant characteristics. Although PT-INR significantly differed between the unmatched groups ( *P* = 0.033), this difference was no longer significant in PSM-1 (27 patients per group). However, in PSM-2 (27 patients per group), the difference in PT-INR remained statistically significant ( *P* = 0.042). Other baseline characteristics showed no significant differences.

**Table:** **Table 1** Patient baseline characteristics.

	**Unmatched**			**PSM cohort-1**			**PSM cohort-2**		
	**SAPH group (n = 27)**	**Control group (n = 227)**	***P* value **	**SAPH group (n = 27)**	**Control group (n = 27)**	***P* value **	**SAPH group (n = 27)**	**Control group (n = 27)**	***P* value **
Sex (male/female)	18/9	135/92	0.537	18/9	20/7	0.776	18/9	21/6	0.544
Age, median (IQR), years	77 (72–83)	77 (69–84)	0.724	77 (72–83)	75 (71–79.5)	0.396	77 (72–83)	76.0 (69.0–82.5)	0.411
Antithrombotic agent usage	8 (29.6%)	61 (26.9%)	0.820	8 (29.6%)	7 (25.9%)	1	8 (29.6%)	8 (29.6%)	1
Resumption of antithrombotic agents after EST			0.750			1			1
Not prescribed	19 (70.4%)	166 (73.1%)		19 (70.4%)	20 (74.1%)		19 (70.4%)	19 (70.4%)	
Within 3 days	8 (29.6%)	58 (25.6%)		8 (29.6%)	7 (25.9%)		8 (29.6%)	8 (29.6%)	
≥ 4 days	0 (0%)	3 (1.3%)		0 (0%)	0 (0%)		0 (0%)	0 (0%)	
Platelet count, median (IQR), (×104/µL)	21.3(16.5–31.3)	21.2(16.5–27.8)	0.593	21.3(16.5–31.3)	26.2(20.7–33.8)	0.268	21.3(16.5–31.3)	21.7(16.6–28.9)	0.876
PT-INR, median (IQR)	1.00(0.95–1.06)	1.05(0.98–1.16)	0.033	1.00(0.95–1.06)	1.02(0.96–1.14)	0.416	1.00(0.95–1.06)	1.07(0.99–1.15)	0.042
Liver cirrhosis	1 (3.7%)	6 (2.6%)	0.549	1 (3.7%)	2 (7.4%)	1	1 (3.7%)	1 (3.7%)	1
Hemodialysis	1 (3.7%)	4 (1.8%)	0.433	1 (3.7%)	1 (3.7%)	1	1 (3.7%)	0 (0%)	1
Concomitant cholangitis	16 (59.3%)	136 (59.9%)	1	16 (59.3%)	13 (48.1)	0.586	16 (59.3%)	10 (37.0%)	0.173
Surgically altered anatomy	1 (3.7%)	8 (3.5%)	1	1 (3.7%)	1 (3.7%)	1	1 (3.7%)	0 (0%)	1
Periampullary diverticulum	11 (40.7%)	66 (29.2%)	0.268	11 (40.7%)	7 (25.9%)	0.387	11 (40.7%)	13 (44.4%)	0.785
Duodenal stricture	2 (7.4%)	12 (5.3%)	0.650	2 (7.4%)	1 (3.7%)	1	2 (7.4%)	1 (3.7%)	1
Primary disease			0.118			0.109			0.438
Choledocholithiasis	12 (44.4%)	150 (66.1%)		12 (44.4%)	17 (63.0%)		12 (44.4%)	17 (63.0%)	
Benign biliary stricture	0 (0%)	3 (1.3%)		0 (0%)	2 (7.4%)		0 (0%)	0 (0%)	
Malignant biliary stricture	13 (48.1%)	66 (29.1%)		13 (48.1%)	8 (29.6%)		13 (48.1%)	9 (33.3%)	
Others	2 (7.4%)	8 (3.5%)		2 (7.4%)	0 (0%)		2 (7.4%)	1 (3.7%)	
IQR, interquartile range; PSM, propensity score matched; PT-INR, prothrombin time-international normalized ratio; SAPH, self-assembling peptide hydrogel.									

Table 2 lists details of the procedure. Mean procedure time was significantly longer in the SAPH group than in the control group (47.0 min vs. 39.5 min, *P* = 0.048) in the unmatched analysis. After adjustment, the difference was no longer significant in either PSM-1 (47.0 min vs. 39.0 min, *P* = 0.130) or PSM-2 (47.0 min vs 34.0 min, *P* = 0.085). Difficult cannulation, procedure content, and number of patients undergoing EST and subsequent EPBD did not significantly differ between the two groups.

**Table:** **Table 2** Procedure details of endoscopic sphincterotomy and related interventions.

	**Unmatched**			**PSM cohort-1**			**PSM cohort-2**		
	**SAPH group (n = 27)**	**Control group (n = 227)**	***P* value **	**SAPH group (n = 27)**	**Control group (n = 27)**	***P* value **	**SAPH group (n = 27)**	**Control group (n = 27)**	***P* value **
Procedure time, median (IQR), min	47.0(35.0–64.0)	39.5(27.0–55.8)	0.048	47.0(35.0–64.0)	39.0(29.5–54.0)	0.130	47.0(35.0–64.0)	34.0(24.0–60.0)	0.085
Combination with papillary dilation	2 (7.4%)	29 (12.8%)	0.548	2 (7.4%)	2 (7.4%)	1	2 (7.4%)	2 (7.4%)	1
Difficult cannulation	15 (55.6%)	128 (56.4%)	1	15 (55.6%)	16 (59.3%)	1	15 (55.6%)	14 (51.9%)	1
Reason for EST			0.525			1			0.763
EST only	1 (3.7%)	5 (2.2%)		1 (3.7%)	1 (3.7%)		1 (3.7%)	0 (0%)	
Stone removal	12 (44.4%)	122 (53.7%)		12 (44.4%)	13 (48.1%)		12 (44.4%)	11 (40.7%)	
Plastic stent placement	9 (33.3%)	71 (31.3%)		9 (33.3%)	8 (29.6%)		9 (33.3%)	12 (44.4%)	
SEMS placement	5 (18.5%)	29 (12.8%)		5 (18.5%)	5 (18.5%)		5 (18.5%)	4 (14.8%)	
EST, endoscopic sphincterotomy; IQR, interquartile range; PSM, propensity score matched; SAPH, self-assembling peptide hydrogel; SEMS, self-expandable metallic stent.									

Incidence of EST-related bleeding was 10.6% (27/254). Among the 27 patients in the SAPH group, 17 had hemorrhage immediately after EST and 10 during subsequent procedures ( Table 3 ). Oozing bleeding occurred in 26 cases, whereas spurting bleeding occurred in one case with stable vital signs. In the SAPH group, 26 of 27 cases (96.3%) achieved successful hemostasis with SAPH. Mean amount of SAPH was 1.5 mL. In one case of spurting bleeding, successful hemostasis could not be achieved with SAPH application alone, requiring SEMS placement as an additional hemostatic technique for successful hemostasis.

**Table:** **Table 3** Background characteristics and procedure outcomes of patients with EST-related hemorrhage.

	**Hemorrhage immediately after EST (n = 18)**	**Hemorrhage occurred by contact with devices (n = 9)**	**Overall (n = 27)**
Antithrombotic agent usage	5 (27.8%)	3 (33.3%)	8 (29.6%)
Resumption of antithrombotic agents after EST			
Not prescribed	13 (72.2%)	6 (66.7%)	19 (70.4%)
Within 3 days	5 (27.8%)	3 (33.3%)	8 (29.6%)
≥ 4 days	0 (0%)	0 (0%)	0 (0%)
Platelet count, median (IQR), (×104/µL)	23.9 (19.0–30.0)	17.7 (14.3–33.1)	21.3 (16.5–31.3)
PT-INR, median (IQR)	0.96 (0.94–1.03)	1.03 (1.00–1.17)	1.00 (0.95–1.06)
Liver cirrhosis	1 (5.6%)	0 (0%)	1 (3.7%)
Hemodialysis	0 (0%)	1 (11.1%)	1 (3.7%)
Concomitant cholangitis	11 (61.1%)	5 (55.6%)	16 (59.3%)
Surgically altered anatomy	1 (5.6%)	0 (0%)	1 (3.7%)
Periampullary diverticulum	8 (44.4%)	3 (33.3%)	11 (40.7%)
Reason for EST			
EST only	1 (5.6%)	0 (0%)	1 (3.7%)
Stone removal	5 (27.8%)	7 (77.8%)	12 (44.4%)
Plastic stent placement	7 (38.9%)	2 (22.2%)	9 (33.3%)
SEMS placement	5 (27.8%)	0 (0%)	5 (18.5%)
Bleeding severity			
Mild	17 (94.4%)	9 (100%)	26 (96.3%)
Moderate	0 (0%)	0 (0%)	0 (0%)
Severe	1 (5.6%)	0 (0%)	1 (3.7%)
Bleeding type			
Miled oozing	9 (50.0%)	7 (77.8%)	16 (59.3%)
Moderate oozing	8 (44.4%)	2 (22.2%)	10 (37.0%)
Spurting	1 (5.6%)	0 (0%)	1 (3.7%)
Technical success of hemostasis using SAPH	17 (94.4%)	9 (100%)	26 (96.3%)
Amount of SAPH, median (IQR), mL	1.45 (1.05–2.00)	1.5 (1.00–2.00)	1.5 (1.00–2.00)
Delayed bleeding	0 (0%)	0 (0%)	0 (0%)
Other adverse events	2 (11.1%)	0 (0%)	2 (7.4%)
EST, endoscopic sphincterotomy; IQR, interquartile range; PT-INR, prothrombin time-international normalized ratio; SAPH, self-assembling peptide hydrogel; SEMS, self-expandable metallic stent.			

Of 254 cases, four (1.57%) experienced delayed bleeding, all in the control group. Although no significant statistical difference was noted between the two groups, none in the SAPH group had delayed bleeding. A subgroup analysis excluding patients with planned SEMS placement (34 patients) was conducted on 220 patients (22 in the SAPH group and 198 in the control group); results revealed that four (1.82%) suffered from delayed bleeding, also all in the control group. Supplementary Table 1 summarizes clinical characteristics, diagnostic criteria, management, and outcomes of these four patients.

Other AEs that occurred in the SAPH group included pancreatitis in two patients, both of which were treated conservatively. In the control group, AEs included pancreatitis, cholangitis, and others in seven, six, and four patients, respectively, all of which were graded as mild or moderate. No significant statistical difference was noted between the two groups or after PSM and at the subgroup analysis ( Table 4 and Table 5 ).

**Table:** **Table 4** Details of adverse events in the full analysis set.

	**Unmatched**			**PSM cohort-1**			**PSM cohort-2**		
	**SAPH group (n = 27)**	**Control group (n = 227)**	***P* value **	**SAPH group (n = 27)**	**Control group (n = 27)**	***P* value **	**SAPH group (n = 27)**	**Control group (n = 27)**	***P* value **
Delayed bleeding	0 (0%)	4 (1.8%)	1	0 (0%)	0 (0%)	NA	0 (0%)	0 (0%)	NA
Pancreatitis	2 (7.4%)	7 (3.1%)	0.246	2 (7.4%)	1 (3.7%)	1	2 (7.4%)	2 (7.4%)	1
Cholangitis	0 (0%)	6 (2.6%)	1	0 (0%)	0 (0%)	NA	0 (0%)	0 (0%)	1
Others	0 (0%)	4 (1.8%)	1	0 (0%)	0 (0%)	NA	0 (0%)	0 (0%)	NA
Overall	2 (7.4%)	21 (9.3%)	1	2 (7.4%)	1 (3.7%)	1	2 (7.4%)	2 (7.4%)	1
NA; not available; PSM, propensity score matched; SAPH, self-assembling peptide hydrogel.									

**Table:** **Table 5** Details of adverse events in the subgroup analysis.

	**Unmatched**			**PSM cohort-1**			**PSM cohort-2**		
	**SAPH group (n = 27)**	**Control group (n = 227)**	***P* value **	**SAPH group (n = 27)**	**Control group (n = 27)**	***P* value **	**SAPH group (n = 27)**	**Control group (n = 27)**	***P* value **
Delayed bleeding	0 (0%)	4 (2.0%)	1	0 (0%)	0 (0%)	NA	0 (0%)	1 (4.8%)	1
Pancreatitis	1 (4.5%)	6 (3.0%)	0.527	1 (4.5%)	0 (0%)	1	1 (4.8%)	0 (0%)	1
Cholangitis	0 (0%)	6 (3.0%)	1	0 (0%)	2 (9.1%)	0.488	0 (0%)	0 (0%)	NA
Others	0 (0%)	3 (1.5%)	1	0 (0%)	1 (4.5%)	1	0 (0%)	1 (4.8%)	1
Overall	1 (4.5%)	19 (9.6%)	0.701	1 (4.5%)	3 (13.6%)	0.607	1 (4.8%)	2 (9.5%)	1
NA, not available; PSM, propensity score matched; SAPH, self-assembling peptide hydrogel.									

## Discussion

This prospective pilot cohort study highlights several important aspects of SAPH application in EST-related hemorrhage. Most notably, not only was the clinical success rate of initial hemostasis using SAPH high, at 96.3%, but also none of those who received SAPH experienced delayed bleeding. Although not achieving statistical significance, our results suggest a trend toward reduced rates of delayed bleeding in SAPH application, indicating a potential prophylactic effect against delayed bleeding. Furthermore, other AEs showed no significant difference between the SAPH and control groups, indicating that SAPH is safe and feasible for EST-related bleeding.

Overall incidence of EST-related bleeding in our cohort was 10.6%, which seems higher than that reported in the study by Ogura et al. (4.7%) 23 . The definition of EST-related bleeding largely explains this discrepancy. Give that the primary endpoint of our study was incidence of delayed bleeding, EST-related hemorrhage was defined as either immediate bleeding after EST or bleeding caused by subsequent device contact with the sphincterotomy site. In the study by Ogura et al. 23 , only immediate post-EST bleeding events were likely included. When our cohort considered only immediate bleeding events, incidence was 7.1% (18/265 cases), which is comparable to previous reports using a similar definition (9.4%-11.9%) 2 6 . Moreover, some device-related bleeding events in our study occurred during subsequent interventions performed by trainees; this observation may also have contributed to the higher overall incidence.

Despite this broader definition, SAPH alone achieved primary hemostasis in 96.3% of our patients, demonstrating excellent hemostatic efficacy. This result is consistent with previous reports showing that SAPH’s hemostatic efficacy for EST-related bleeding is comparable to that of conventional methods such as balloon tamponade 2 13 23 , epinephrine injection 2 13 24 , and SEMS placement 24 , with a clinical success rate of 77.2% to 100%. Moreover, SAPH efficacy and safety have been demonstrated not only for EST-related hemorrhage 23 but also for other gastrointestinal tract bleeding cases in previous studies 10 11 25 26 . In the current study, a broader definition of EST-related hemorrhage was adopted to include all bleeding types, including spurting, to evaluate the primary hemostatic effect of SAPH across the full spectrum of bleeding severity. SAPH offers the advantages of transparency, which allows the examiner to continuously visualize the bleeding point, and easy removability, which prevents interference with subsequent hemostatic interventions. Indeed, in our cohort, SAPH application facilitated secondary hemostasis (e.g., SEMS placement) following spurting bleeding reduction. Therefore, its use as a first-line treatment choice for EST-related hemorrhage may be attributed to not only its high hemostatic efficacy but also to its unique physical property as a transparent agent, which aids in identifying the bleeding site without interfering with subsequent hemostatic procedures 12 26 . Taken together, SAPH may serve as a promising first-line option, even in severe bleeding cases.

Of note, despite applying SAPH only to patients with EST-related hemorrhage in our study, which is a known risk factor for delayed bleeding 9 20 27 , none of those in the SAPH group experienced delayed bleeding. Although our study did not reach statistical significance, the result is promising in terms of preventing delayed bleeding. A previous retrospective study by Inoue et al. reported significant reductions in delayed bleeding rates with SAPH application 17 . In addition, the potential of SAPH to prevent delayed bleeding has been demonstrated in other gastrointestinal bleeding settings 16 25 28 . Thus, the prophylactic effect of SAPH on delayed bleeding may extend beyond EST-related hemorrhage, potentially offering broader applications in gastrointestinal endoscopy. Further research with a large sample size might reveal a statistically significant preventive effect of SAPH against delayed bleeding.

Regarding other AEs, no significant difference was demonstrated between the SAPH and control groups, consistent with previous reports. The hemostatic mechanism of SAPH is that a hydrogel matrix acts as a mechanical barrier that covers the bleeding site to provide a hemostatic effect 10 29 . Furthermore, a preclinical study showed that the hydrogel matrix facilitated cell and tissue retention during healing, and some recent studies revealed that SAPH promotes mucosal regeneration 11 28 30 . However, these characteristics of SAPH raise the concern that acute pancreatitis or cholangitis may occur because the pancreatic duct or bile duct opening could be obstructed mechanically or histologically. Nevertheless, the hydrogel matrix may cover the bleeding point at a microscopic level, although it does not mechanically obstruct the pancreatic duct or bile duct opening because of its easy removal. This concept may help explain why SAPH application did not significantly alter the rates of other AEs.

This study has several limitations, including its nonrandomized design and small sample size. Another important limitation is that SAPH was applied only in patients with EST-related bleeding, whereas the control group comprised nonbleeders. Under current Japanese insurance regulations, SAPH is approved solely for use in active bleeding cases; therefore, applying it to nonbleeders was not ethically permissible. However, this study design inherently limits the ability to draw causal inference regarding SAPH’s preventive effect on delayed bleeding. In addition, given that this research is a pilot study, no a priori sample size or power calculation was performed and only four delayed bleeding events were examined. These factors limit the statistical strength of our findings; thus, the results should be considered exploratory. Finally, all EST procedures were performed by fully trained endoscopists with extensive expertise in ERCP-related procedures; thus, generalizability of these results cannot be guaranteed.

## Conclusions

In conclusion, although limited by its pilot, nonrandomized design, our study demonstrated that SAPH achieved adequate primary hemostatic effect and was safe for management of EST-related hemorrhage. Although the observed absence of delayed bleeding in the SAPH group did not reach statistical significance, this finding is hypothesis-generating and suggests a potential preventive benefit. Future large-scale studies and randomized controlled trials are necessary to validate these preliminary observations and to clarify the role of SAPH in preventing delayed bleeding after EST.

## Supplementary Material

Supplementary Material Zusatzmaterial
