# Supplementary material for: Effect of a self-assembling peptide hydrogel on delayed bleeding following endoscopic sphincterotomy: Prospective pilot cohort study
Source: Endosc Int Open. 2026 Feb 25;14:a28033921. doi: 10.1055/a-2803-3921 (PMC12951035; doi:10.1055/a-2803-3921)
Supplement: Supplementary file 1 — Supplementary Material [file 10-1055-a-2803-3921_28072326.pdf]

Thieme

Supplementary Table 1 Clinical details of patients with delayed bleeding (n = 4).

Case	Age/sex	Primary disease	Indication for EST	Concomitant EPBD	Antithrombotic agent (resumption)	Comorbidities (cirrhosis/ hemodialysis/concomitant cholangitis)	EST-related bleeding
1	68/male	Pancreatic cancer	Plastic stent placement	No	DOAC (1 day)	No/No/Yes	No
2	84/male	Bile duct stone	Plastic stent placement	No	Aspirin (1 day)	No/No/Yes	No
3	70/male	Bile duct stone	Stone removal	12 mm balloon dilation	None	No/No/No	No
4	65/male	Bile duct stone	Stone removal	No	Aspirin (1day)	No/No/Yes	No

Onset of bleeding (day)	Diagnostic criteria	Severity	Management (transfusion/ reintervention)	Clinical course
6	Endoscopic finding at scheduled follow-up (symptomatic)	Mild	PuraStat application	Uneventful; no prolongation of hospital stay
3	Endoscopic finding at scheduled follow-up (symptomatic)	Mild	HSE injection	Uneventful; no prolongation of hospital stay
3	Melena	Moderate	SEMS placement	Hospital stay prolonged by 5 days
3	Melena	Moderate	SEMS placement	Hospital stay prolonged by 4 days

DOAC, direct oral anticoagulant; EPBD, endoscopic papillary balloon dilation; EST, endoscopic sphincterotomy; HSE, hypertonic saline epinephrine; SEMS, self-expandable metallic stent.

Endosc Int Open | DOI 10.1055/a-2803-3921 | © The Author(s).
